# Transcriptome-Wide Analysis and Experimental Validation from FFPE Tissue Identifies Stage-Specific Gene Expression Profiles Differentiating Adenoma, Carcinoma In-Situ and Adenocarcinoma in Colorectal Cancer Progression

**DOI:** 10.3390/ijms26094194

**Published:** 2025-04-28

**Authors:** Faisal Alhosani, Reem Sami Alhamidi, Burcu Yener Ilce, Alaa Muayad Altaie, Nival Ali, Alaa Mohamed Hamad, Axel Künstner, Cyrus Khandanpour, Hauke Busch, Basel Al-Ramadi, Rania Harati, Kadria Sayed, Ali AlFazari, Riyad Bendardaf, Rifat Hamoudi

**Affiliations:** 1 Research Institute of Medical and Health Sciences, University of Sharjah, Sharjah P.O. Box 27272, United Arab Emirates; u20105895@sharjah.ac.ae (F.A.); ralhamidi@sharjah.ac.ae (R.S.A.); bilce@sharjah.ac.ae (B.Y.I.); alaa.abed@sharjah.ac.ae (A.M.A.); nevalalmaleh@gmail.com (N.A.); rharati@sharjah.ac.ae (R.H.); 2 Department of Clinical Sciences, College of Medicine, University of Sharjah, Sharjah P.O. Box 27272, United Arab Emirates; 3 Medical Systems Biology Group, Lübeck Institute of Experimental Dermatology, University of Lübeck, Ratzeburger Allee 160, 23538 Lübeck, Germany; axel.kuenstner@uni-luebeck.de (A.K.); hauke.busch@uni-luebeck.de (H.B.); 4 Forensic Laboratory Department, Sharjah Police Headquarters, Sharjah P.O. Box 1965, United Arab Emirates; 5 College of Health Sciences, Abu Dhabi University, Abu Dhabi P.O. Box 59911, United Arab Emirates; alaa.mohamed@adu.ac.ae; 6 Department of Hematology and Oncology, University Cancer Center Schleswig-Holstein, University Hospital Schleswig-Holstein, University of Lübeck, 23562 Lübeck, Germany; cyrus.khandanpour@uksh.de; 7 Department of Medical Microbiology and Immunology, College of Medicine and Health Sciences, United Arab Emirates University, Al Ain P.O. Box 15551, United Arab Emirates; ramadi.b@uaeu.ac.ae; 8 Zayed Center for Health Sciences, United Arab Emirates University, Al Ain P.O. Box 15551, United Arab Emirates; 9 ASPIRE Precision Medicine Research Institute Abu Dhabi, United Arab Emirates University, Al Ain P.O. Box 15551, United Arab Emirates; 10 Department of Pharmacy Practice and Pharmacotherapeutics, College of Pharmacy, University of Sharjah, Sharjah P.O. Box 27272, United Arab Emirates; 11 Department of Pathology and Laboratory Medicine, American Hospital Dubai, Dubai P.O. Box 3050, United Arab Emirates; ksayed@ahdubai.com; 12 Mediclinic Welcare Hospital, Dubai P.O. Box 31500, United Arab Emirates; ali.alfzari@mediclinic.ae; 13 Oncology Unit, University Hospital Sharjah, Sharjah P.O. Box 72772, United Arab Emirates; riyad.bendardf@uhs.ae; 14 Center of Excellence for Precision Medicine, Research Institute of Medical and Health Sciences, University of Sharjah, Sharjah P.O. Box 27272, United Arab Emirates; 15 BIMAI-Lab, Biomedically Informed Artificial Intelligence Laboratory, University of Sharjah, Sharjah P.O. Box 27272, United Arab Emirates; 16 ASPIRE Precision Medicine Research Institute Abu Dhabi, University of Sharjah, Sharjah P.O. Box 27272, United Arab Emirates; 17 Division of Surgery and Interventional Science, University College London, London WC1E 6BT, UK

**Keywords:** colorectal cancer (CRC), adenoma, adenocarcinoma, tumor immune microenvironment, GSEA, biomarker discovery

## Abstract

Colorectal cancer (CRC) progression occurs through three stages: adenoma (pre-cancerous lesion), carcinoma in situ (CIS) and adenocarcinoma, with tumor stage playing a pivotal role in the prognosis and treatment outcomes. Despite therapeutic advancements, the lack of stage-specific biomarkers hinders the development of accurate diagnostic tools and effective therapeutic strategies. This study aims to identify stage-specific gene expression profiles and key molecular mechanisms in CRC providing insights into molecular alterations across disease progression. Our methodological approach integrates the use of absolute gene set enrichment analysis (absGSEA) on formalin-fixed paraffin-embedded (FFPE)-derived transcriptomic data, combined with large-scale clinical validation and experimental confirmation. A comparative whole transcriptomic analysis (RNA-seq) was performed on FFPE samples including adenoma (*n* = 10), carcinoma in situ (CIS) (*n* = 8) and adenocarcinoma (*n* = 11) samples. Using absGSEA, we identified significant cellular pathways and putative molecular biomarkers associated with each stage of CRC progression. Key findings were then validated in a large independent CRC patient cohort (*n* = 1926), with survival analysis conducted from 1336 patients to assess the prognostic relevance of the candidate biomarkers. The key differentially expressed genes were experimentally validated using real-time PCR (RT-qPCR). Pathway analysis revealed that in CIS, apoptotic processes and Wnt signaling pathways were more prominent than in adenoma samples, while in adenocarcinoma, transcriptional co-regulatory mechanisms and protein kinase activity, which are critical for tumor growth and metastasis, were significantly enriched compared to adenoma. Additionally, extracellular matrix organization pathways were significantly enriched in adenocarcinoma compared to CIS. Distinct gene signatures were identified across CRC stages that differentiate between adenoma, CIS and adenocarcinoma. In adenoma, *ARRB1*, *CTBP1* and *CTBP2* were overexpressed, suggesting their involvement in early tumorigenesis, whereas in CIS, *RPS3A* and *COL4A5* were overexpressed, suggesting their involvement in the transition from benign to malignant stage. In adenocarcinoma, *COL1A2*, *CEBPZ*, *MED10* and *PAWR* were overexpressed, suggesting their involvement in advanced disease progression. Functional analysis confirmed that *ARRB1* and *CTBP1*/2 were associated with early tumor development, while *COL1A2* and *CEBPZ* were involved in extracellular matrix remodeling and transcriptional regulation, respectively. Experimental validation with RT-qPCR confirmed the differential expression of the candidate biomarkers (*ARRB1*, *RPS3A*, *COL4A5*, *COL1A2* and *MED10)* across the three CRC stages reinforcing their potential as stage-specific biomarkers in CRC progression. These findings provide a foundation to distinguish between the CRC stages and for the development of accurate stage-specific diagnostic and prognostic biomarkers, which helps in the development of more effective therapeutic strategies for CRC.

## 1. Introduction

Colorectal cancer (CRC) is the third most common cancer worldwide and the second leading cause of cancer-related deaths. CRC is mostly detected at advanced stages limiting treatment options and contributing to high mortality rates. According to the International Agency for Research on Cancer (IARC), the estimated number of CRC cases globally was 1.93 million in 2022 [1]. Most CRC cases develop sporadically via the adenoma–carcinoma in situ–adenocarcinoma sequence, a progression driven by genetic and epigenetic changes that disrupt normal epithelial renewal and drive tumorigenesis [2]. Despite established biomarkers such as the *APC* and *KRAS* genes, their utility in distinguishing CRC stages is limited, necessitating further molecular investigations.

CRC progresses primarily through two major pathways: the conventional adenoma–carcinoma sequence and the serrated pathway [3]. In the conventional adenoma–carcinoma sequence, CRC progresses through three primary stages: adenoma, carcinoma in situ (CIS) and adenocarcinoma. Adenomas are benign tumors that originate from glandular colon epithelium and represent the pre-cancerous stage of CRC [4]. They can be classified histologically into tubular (the most common type, characterized by tubular structures), tubulovillous (comprising both tubular and villous structures, with an elevated risk of progression to cancer) and villous types (the least common but associated with a significant risk of malignancy). *APC* is a widely utilized biomarker for the detection of adenomatous colon polyps, and the *MUTYH* mutation has the potential to diagnose a distinct type of rare colorectal adenoma (*MUTYH*-associated polyposis (MAP)) [5,6]. CIS is a localized, non-invasive stage where abnormal cell growth is confined to the mucosa. If left untreated, CIS can progress to an invasive form of carcinoma. CIS is often asymptomatic and typically detected during colonoscopy examinations. Adenocarcinoma, the invasive stage of CRC, arises from glandular epithelial cells and has the potential for metastasis [7,8,9]. *KRAS*, *BRAF*, *NRAS* and *PIK3CA* gene mutations serve as prognostic biomarkers for CRC. Currently, methylated *MLH-1*, *VIM*, and *SEPT9* are being developed as diagnostic tools for detecting colon carcinomas [10,11]. The serrated pathway, on the other hand, involves sessile serrated lesions (SSLs) and traditional serrated adenomas (TSAs), which can also progress to carcinoma through distinct molecular and histopathological mechanisms [3].

In clinical practice, colorectal cancer staging is conventionally defined by the TNM classification system (stages 0 to IV), where stage 0 represents the earliest stage, described as CIS and confined to the epithelium or lamina propria. Stage I is characterized by tumor infiltration in the mucosa and submucosa or beyond the submucosal layers where tumors may gain access to the lymphovascular system. Stages II and III indicate progressive invasion into deeper tissues and lymph node involvement, and stage IV denotes distant metastasis [12]. Thus, adenomas typically correspond to precancerous lesions preceding stage I, carcinoma in situ aligns closely with early-stage tumors (stage 0 or I) and adenocarcinomas include invasive cancers that range broadly across stages I–IV depending on their invasiveness and metastatic potential. CRC is also classified based on the molecular makeup of the tumor. The molecular classification system for colorectal cancer, known as the Consensus Molecular Subtypes (CMSs), classifies CRC into four distinct subtypes: CMS1 (MSI immune), CMS2 (canonical), CMS3 (metabolic) and CMS4 (mesenchymal), each with unique molecular characteristics, clinical behavior and treatment responses [13,14].

During CRC development, the clinical symptoms across the three stages of the conventional CRC pathways development (adenoma–CIS–adenocarcinoma) may overlap, including changes in bowel habits, abdominal pain, weight loss and rectal bleeding [15]. This overlap highlights the need for molecular investigations to identify unique stage-specific signatures.

Accurate diagnostic methods are hindered by tumor heterogeneity and the extensive number of genetic mutations associated with CRC, making testing numerous genes impractical [16]. Researchers suggest that individuals over 20 years of age presenting with symptoms such as abdominal masses, progressive weight loss, unexplained anemia, blood in stool, altered bowel habits or persistent abdominal discomfort require further clinical investigation [17]. Patients with suspected CRC are diagnosed through different methods. These include positron emission computed tomography (PET), which provides information on a tumor’s metabolic attributes and the anatomical site [18]. Computed tomography (CT) and Magnetic Resonance Imaging (MRI) are commonly used for CRC staging to display the underlying conditions, such as abdominal lymph nodes, lesion sizes, and impact of lesions on proximal organs and tissues [19]. Transabdominal Ultrasound is used to detect abdominal lymph nodes and intestinal masses [20]. Colorectal endoscopy provides direct observation of lesion shape, size and position [21,22]. Fecal occult blood tests are used to check for hidden blood in stool, with positive results for approximately 5 mL of colorectal bleeding [23]. These methods are valuable but insufficient in identifying reliable stage-specific biomarkers.

The diagnosis and treatment of CRC remain challenging due to the absence of clear and definitive biomarkers for specific stages, thereby limiting early detection and personalized treatment strategies [24,25,26]. This study aims to address this gap by identifying and validating molecular biomarkers that distinguish between adenomas, CIS and Adenocarcinomas in CRC progression considering the conventional pathway of CRC. To achieve this, we employed a custom in-house bioinformatics pipeline to uncover distinct transcriptomic profiles and identify differentially expressed genes in adenoma, CIS and adenocarcinomas. The potential biomarkers were then experimentally validated through RT-qPCR analysis using RNAs extracted from FFPE samples of colorectal adenoma, carcinoma in situ and adenocarcinoma patients.

## 2. Results

### 2.1. Differential Gene Expression Signatures Across Adenoma, CIS and Adenocarcinoma Stages in CRC Patients

**Figure 1:**
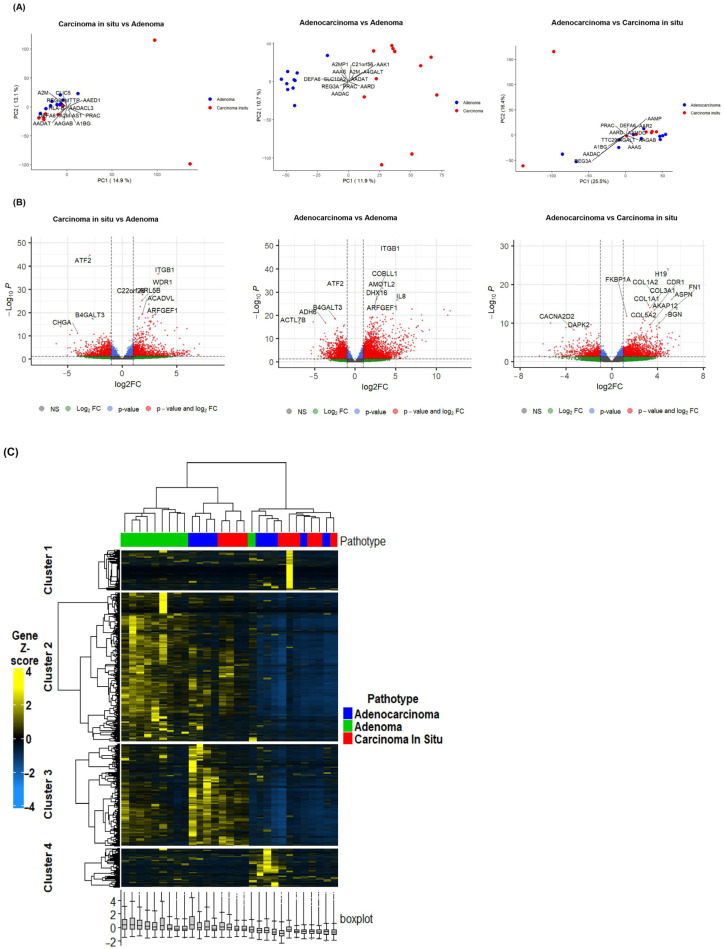
Principal component analysis (PCA), volcano plot and heatmap of DEGs. (**A**) PCA plots showing the separation between each group comparison: carcinoma in situ vs. adenoma (left), adenocarcinoma vs. adenoma (middle), and adenocarcinoma vs. carcinoma in situ (right). (**B**) Volcano plot of DEGs between each group comparison. The X-axis represents the Log 2-fold change, and the Y-axis represents the adjusted *p*-values. Genes significantly up- or downregulated (log2FC, *p* < 0.05) are shown in red. The correlation coefficient between adenoma and adenocarcinoma DEGs is r: 0.814, suggesting a strong positive correlation between the gene expression levels in the two groups. (**C**) Heatmap of the CRC stages using unsupervised hierarchical clustering.

Principal component analysis (PCA) of the RNASeq data is represented in Figure 1A. Comparing CIS and adenoma samples, the PCA plot showed partial overlapping profiles between CIS and adenoma samples, suggesting that these CRC stages share transcriptomic profiles. A better separation was observed between adenocarcinoma and adenoma samples but with minimal overlap. The PCA plot for CIS and adenocarcinoma samples showed partial overlap between these two stages, suggesting that while they may have distinct gene expression profiles, some similarities exist (Figure 1A). Transcriptomic analysis identified distinct gene expression profiles between the three stages (4546 significant DEGs between CIS and adenoma, 6738 significant DEGs between adenocarcinoma and adenoma, and 3245 between adenocarcinoma and CIS). Figure 1B represents the volcano plots for each group comparison. The parameters selected to define the DEGs for downstream analysis were *p*-value < 0.05 and log2FC > 1.5 and <−1.5. The full list of DEGs is shown in Supplementary Table S1.

The heatmap of the CRC subgroups using unsupervised hierarchical clustering is represented in Figure 1C. This hierarchical clustering heatmap identified four distinct clusters. Cluster 1 reveals relatively uniform expression signatures across all CRC stages. Cluster 2 reveals genes that are highly expressed in adenoma but diminish in later stages; this may reflect early-stage specificity. Cluster 3 demonstrates there is a gradual shift in gene expression from adenoma to later stages, pointing to transitional regulatory activity. Cluster 4 shows genes with variable expression across stages, with selective genes highly expressed in a subset of adenocarcinoma or CIS samples, indicating stage-specific heterogeneity. The heatmap showed that while most adenocarcinoma samples displayed distinct expression profiles, a subset clustered closely with adenoma samples. This finding suggests that a subset of adenocarcinoma tumors may retain molecular signatures characteristic of earlier stages of CRC progression, indicating a heterogeneity within this stage. Such intra-stage heterogeneity emphasizes the complexity of CRC progression and may be driven by mutations in key driver genes such as *APC*, *KRAS*, *BRAF*, *PIK3CA*, or TP53, each known to profoundly influence tumor biology and progression patterns. These genetic alterations could account for the diverse expression patterns observed, with potential implications for diagnosis, prognosis and personalized treatment strategies.

### 2.2. Gene-Set Enrichment Analysis of Differentially Expressed Genes Highlights Distinct Molecular Signatures Between Adenoma, CIS and Adenocarcinoma

Absolute (absGSEA) and Standard GSEA were conducted as described [27] to identify significantly enriched pathways in the three CRC stages utilizing the MSigDB database that encompasses diverse gene sets from the C1 to C8 collections (Table 1). Emphasis was placed on the C5 and C7 as they offer detailed insights into molecular, biological and immunological signatures. Using a nominal *p*-value threshold of <0.05 and a False Discovery Rate (FDR) of *q*-value < 0.25, 99 and 1208 significant pathways from C5 and C7 were identified, respectively. The enriched pathways demonstrate diverse biological processes and molecular functions involving signal transduction, transcription regulation, catalytic activity and molecular binding. The full list of significant pathways from each collection is provided in Supplementary Materials Tables S2–S4. After identifying the significant pathways, gene frequency within these significant pathways was analyzed to assess their level of association and relevance to different stages of CRC. Supplementary Materials Table S5 presents the top 20% selected genes from C5 and C7 gene sets ranked from highest to lowest based on their frequency (*p* < 0.05). The log2 fold change values are oriented towards adenocarcinoma, indicating differences in expression in this condition. Genes with higher frequency are likely to play critical roles in pathways enriched for adenocarcinoma, highlighting their potential importance in the disease’s progression and biological processes.

### 2.3. Gene-Set Enrichment Analysis on Patient Samples Revealed Distinctive Tumor Stage-Mediated Activation of Transcriptional Co-Regulatory Mechanisms, Protein Kinase Functional Pathways and Cellular Metabolic Processes

**Figure 2:**
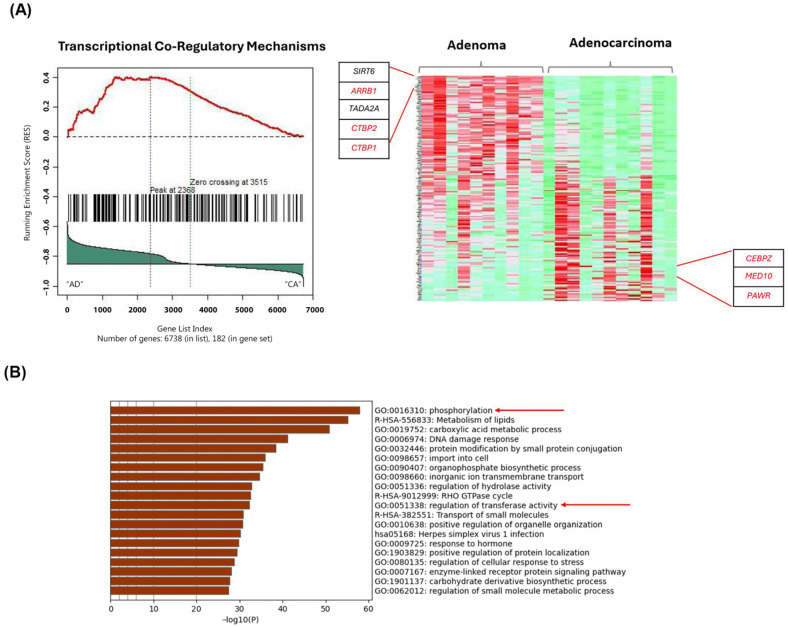
Significantly enriched pathways based on gene frequency obtained from adenoma vs. adenocarcinoma. (**A**) Transcriptional coregulatory mechanism pathway includes genes from the top 20 gene frequency analysis, such as *SIRT6*, *ARRB1*, *TADA2A*, *CTBP2* and *CTBP1* (all downregulated in adenocarcinoma). Genes highlighted in red are the selected putative biomarkers for further validation. (**B**) Enriched pathways identified using Metascape obtained based on gene frequency results ranked from highest to lowest frequency. Aberrant phosphorylation and regulation of transferase activity (indicated by red arrows) critically modulate key signaling molecules in CRC which drives uncontrolled cellular proliferation, resistance to apoptosis and increased metastasis potential.

**Figure 3:**
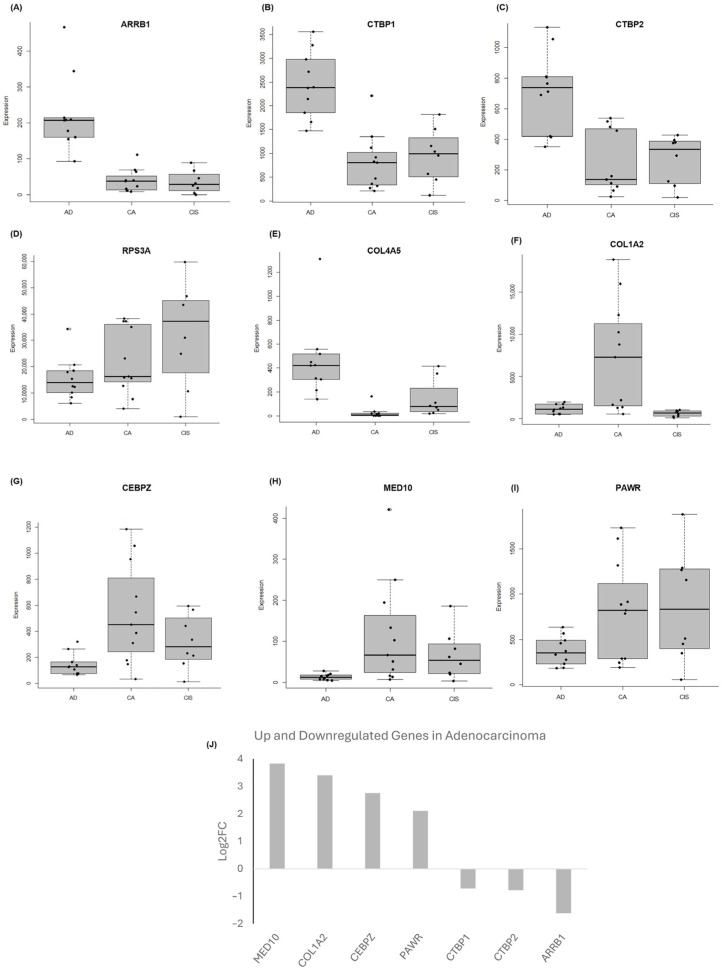
DEGs presented as boxplots revealing distinct differential expression profiles among adenoma, CIS and adenocarcinoma. (**A**) *ARRB1*, (**B**) *CTBP1* and (**C**) *CTBP2* genes exhibited significant upregulation in adenoma, while (**D**) *RPS3A* and (**E**) *COL4A5* exhibited significant upregulation in CIS, and (**F**) *COL1A2*, (**G**) *CEBPZ*, (**H**) *MED10* and (**I**) *PAWR* genes exhibited significant upregulation in adenocarcinoma. These boxplots illustrate differential expression patterns identified through RNA-seq analysis across CRC stages. AD: adenoma; CIS: carcinoma in situ; CA: adenocarcinoma. (**J**) Log2FC of the up- and downregulated biomarkers in adenocarcinoma compared to adenoma, ranked by highest to lowest Log2FC values.

The transcriptional co-regulatory mechanisms pathway was identified, exhibiting DEGs in the adenocarcinoma cohorts compared to adenoma; these genes include *CEBPZ*, *MED10* and *PAWR*. *ARRB1*, *CTBP1* and *CTBP2* were genes identified based on the top 20 frequently occurring genes and are upregulated in adenoma samples (Figure 2A). Furthermore, the affected pathways include those linked with phosphorylation and the regulation of transferase activity pathways as inferred from the enrichment analysis (Figure 2B). Previous studies showed that dysregulation of these pathways is pivotal in CRC cell proliferation and tumorigenesis [28,29,30]. Boxplot graphs in Figure 3A–I present the gene expression differences of the selected biomarkers in adenoma, CIS and adenocarcinoma. The selection criteria for candidate biomarkers were defined based on several factors to ensure statistical robustness and biological relevance. The DEGs were required to meet a statistical significance threshold of *p*-value < 0.05, determined from the gene frequency analysis, with independent validation analysis using an unpaired t-test of unequal variance to confirm the consistency of significance. Additionally, DEGs were filtered based on a log2FC threshold of >1.5 or <−1.5, ensuring biologically meaningful expression differences. To account for the variability inherent in FFPE samples, an additional parameter was implemented: genes exhibiting high expression were required to have an average raw read count of >100 in one group while maintaining a minimum mean threshold of 30 counts in the other group, which represents lower expression. Furthermore, pathway and biological relevance ensured that at least two or more candidate genes were involved in multiple relevant biological pathways for validation. Distinct gene expression profiles are observed especially between adenoma and adenocarcninoma samples. *ARRB1* (*p* = 2.79 × 10^−5^), *CTBP1* (*p* = 1.33 × 10^−5^) and *CTBP2* genes (*p* = 0.0002) exhibited notable upregulation in adenoma, whereas *COL1A2* (*p* = 0.0075), *CEBPZ* (*p* = 0.0057), *MED10* (*p* = 0.0196) and *PAWR* genes (*p* = 0.00215) showed significant upregulation in adenocarcinoma. Figure 3J represents the Log2FC of the up- and downregulated biomarkers in adenocarcinoma.

Subsequent investigation revealed that the *COL1A2* gene (*p*-value: 0.0064), though not ranked among the top 20 frequently occurring genes, emerged as a promising candidate biomarker with potential in distinguishing adenocarcinoma from CIS and adenoma (Supplementary Table S6). In addition, while not ranked as well in the top 20 frequently occurring genes, RPS3A (*p* = 0.048) was a putative biomarker that may distinguish CIS from adenoma.

Several candidate biomarkers exhibited expression patterns suggestive of potential diagnostic relevance. However, these genes were not prioritized for downstream validation due to insufficient data reliability. While further investigation is required to confirm their biological relevance, preliminary observations are provided in Figure S1.

### 2.4. Validation of the Selected Candidate Biomarkers Using RT-qPCR

**Figure 4:**
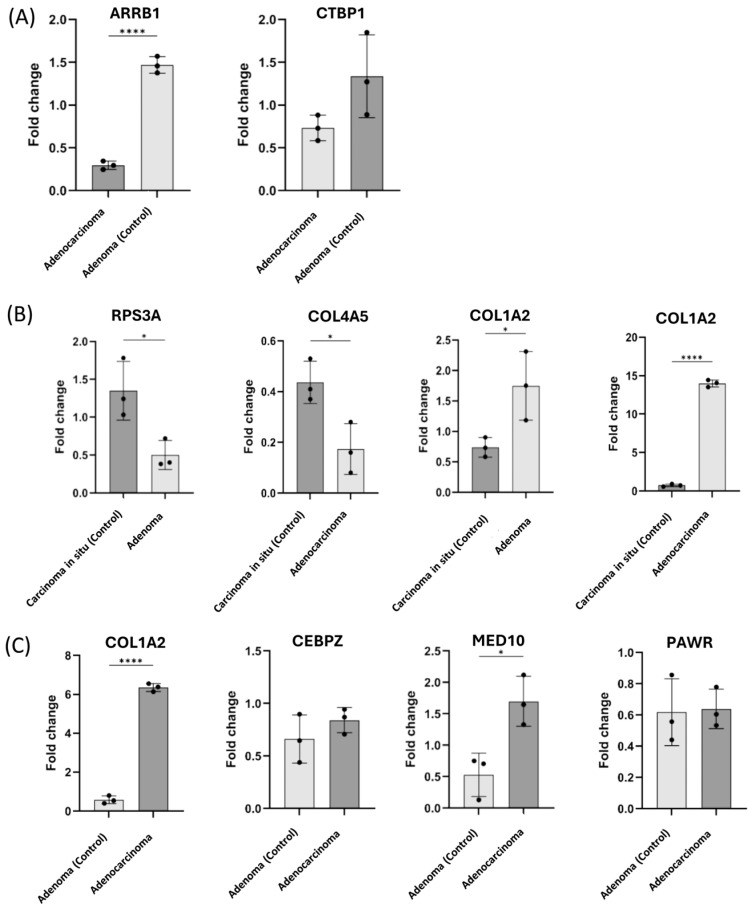
Quantitative real-time PCR of selected genes identified in correlation to enriched pathways (**A**) in adenocarcinoma compared to adenoma (as a control), (**B**) in adenoma and adenocarcinoma compared to CIS (as a control) (**C**) in adenoma (control) compared to adenocarcinoma. Three biological samples in each group were investigated, and each sample was technically replicated three times. The data display the fold change in gene expression between groups. The data were analyzed using an unpaired two-tailed *t* test. A *p*-value ≤ 0.05 was considered significant; * reveals that the *p*-value was <0.05, and **** reveals that the *p*-value was <0.0001.

To validate the findings from absGSEA using the RNAseq data, qRT-PCR was performed on adenoma, adenocarcinoma and CIS samples (Figure 4). *COL1A2* expression was significantly elevated in adenocarcinoma compared to CIS (*p*-value < 0.0001) as well as adenoma compared to CIS (*p*-value < 0.05). *RSP3A* and *COL4A5* were significantly overexpressed in CIS compared to adenoma and adenocarcinoma, respectively (*p*-value < 0.05). Additionally, *COL1A2* (*p*-value < 0.0001) and *MED10* (*p*-value < 0.05) were significantly higher in the adenocarcinoma group compared to the adenoma group. *ARRB1* was also validated as significantly upregulated (*p*-value < 0.0001) in the adenoma samples compared to adenocarcinoma. These findings support the reliability of the RNAseq data and emphasize the potential molecular signatures to distinguish key CRC stages.

### 2.5. Validation of the Candidate Biomarkers Differentiating Between Adenoma, CIS and Adenocarcinoma Using Independent Cohort of CRC Patients

**Figure 5:**
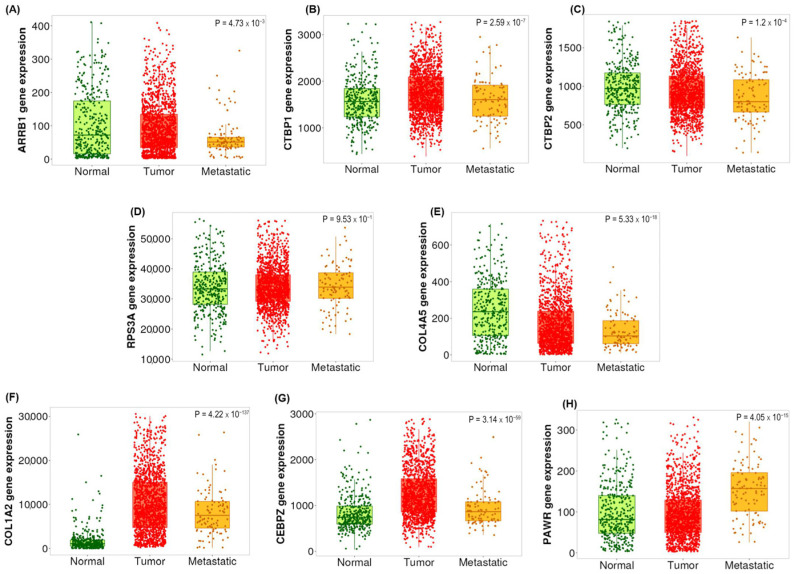
Comparison of expression levels for candidate biomarkers across normal, tumor and metastatic colorectal cancer tissues. Expression profiles were obtained from the TNMplot database. Biomarkers include the following: (**A**) *ARRB1*, (**B**) *CTBP1*, (**C**) *CTBP2*, (**D**) *RPS3A*, (**E**) *COL4A5*, (**F**) *COL1A2*, (**G**) *CEBPZ* and (**H**) *PAWR*. *ARRB1* exhibited similar expression levels between normal and tumor tissues, whereas other genes showed significantly different expression levels across the three conditions. No data were available for *MED10*. Images obtained from TNMplot (https://tnmplot.com/analysis/, accessed on 5 December 2024).

**Figure 6:**
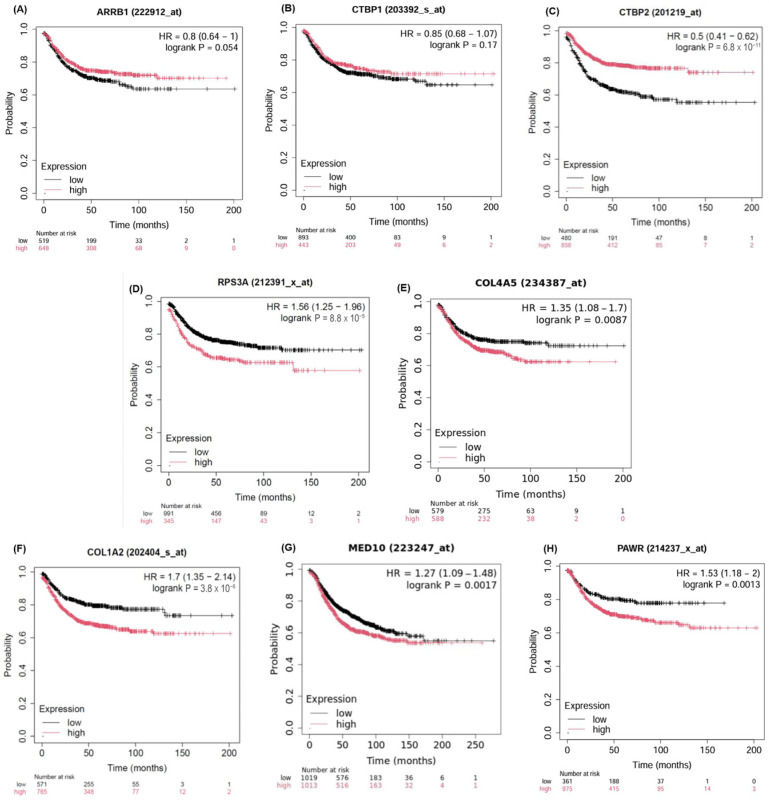
Survival analysis of candidate molecular biomarkers. Adenoma biomarkers: (**A**) *ARRB1*, (**B**) *CTBP1* and (**C**) *CTBP2*; CIS biomarkers: (**D**) *RPS3A* and (**E**) *COL4A5*; and adenocarcinoma biomarkers: (**F**) *COL1A2*, (**G**) *MED10*, and (**H**) *PAWR*. No data were found for CEBPZ.

Following our experimental RT-qPCR validation, we assessed the selected candidate biomarkers further through independent transcriptomic datasets provided by TNMplot (https://tnmplot.com/analysis/, accessed on 5 December 2024), comparing expression across normal, tumor and metastatic colorectal cancer conditions. TNMplot is a web-based tool that allows users to compare gene expression levels across normal, tumor and metastatic tissues using publicly available transcriptomic datasets. Figure 5 reveals significant differences in expression levels of the selected biomarkers across normal, tumor and metastatic conditions obtained from TNMplot (https://tnmplot.com/analysis/, accessed on 5 December 2024). Collectively, the biomarkers exhibited higher expression in the tumor and metastatic states compared to the normal state, except for *ARRB1* (Figure 5A), which exhibited similar expression in both normal and tumor states. This finding highlights that these genes can potentially serve as prognostic biomarkers to distinguish between adenoma and adenocarcinoma and aid in understanding the molecular changes associated with colorectal cancer. Survival analysis (Figure 6) further highlights the clinical relevance of these genes. Higher expression of the adenoma-associated biomarkers (Figure 6A–C) correlates with improved survival outcomes, while higher expression of CIS (Figure 6D,E) and adenocarcinoma biomarkers (Figure 6F–H) is associated with poorer prognosis. Consistent with our transcriptomics findings, *COL1A2*, *CEBPZ* and *PAWR* were upregulated; genes including *ARRB1*, *CTBP1* and *CTBP2* were downregulated in adenocarcinoma (Figure 4). While our RNA-seq analysis did not include metastatic CRC samples, validation using TNMplots revealed that certain biomarkers exhibited increased expression in metastatic tissues compared to normal and tumor tissues. Although this suggests a possible association with advanced disease, these findings should be interpreted with caution, as no metastatic samples were included in our discovery cohort.

### 2.6. Identification of Immune Cell Types in Different Types of CRC Patients

**Figure 7:**
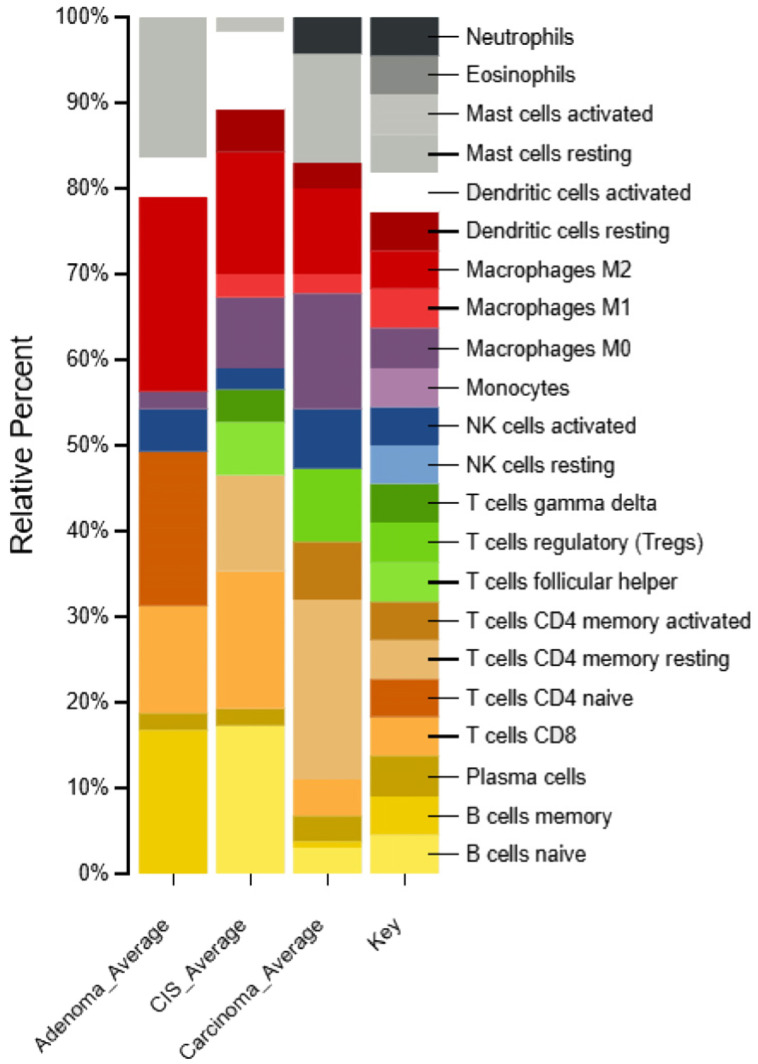
Comparison of CIBERSORTX immune cell fractions between adenoma, CIS and adenocarcinoma samples.

In CRC samples, distinct immune cell profiles were observed across adenoma, CIS and adenocarcinoma (Figure 7 and Supplementary Table S7). The highest fractions of immune cells in adenoma samples compared to CIS and adenocarcinoma were macrophages (23%) followed by naïve CD4^+^ T cells (18%), and memory B cells (17%), consistent with previous findings [31,32,33]. In contrast, resting dendritic cells and M1 macrophages were almost completely depleted in adenoma samples compared to CIS and adenocarcinoma. In CIS samples, the predominant immune cell type was naïve B cells (17%), which may reflect the transitional state from adenoma as previously reported [34]. This was followed by follicular helper T cells (6%) and γδ T cells (4%). These findings suggest the activation of an anti-tumor immune response potentially involving interactions with B cells in mediating humoral immunity [35,36]. Notably, activated mast cells were completely depleted in CIS compared to adenoma and adenocarcinoma. In adenocarcinoma samples, the most abundant immune cells were resting memory CD4^+^ T cells (21%) followed by M0 macrophages (14%), regulatory T cells (8%) and activated memory CD4^+^ T cells (7%) compared to adenoma and CIS as observed previously [37,38,39,40].

## 3. Discussion

To the best of our knowledge, we present the first study to examine the distinct gene expression profiles across adenoma, CIS and adenocarcinoma within a diverse population residing in the UAE (with 29.59% of participants being Emirati nationals). The findings offer valuable insights into the molecular mechanisms as well as immune signatures underlying CRC progression, highlighting unique gene expression profiles and enriched pathways that characterize the transition from adenoma to CIS and ultimately adenocarcinoma.

While numerous studies have explored molecular drivers of CRC progression, few have performed transcriptome-wide profiling on FFPE samples from distinct CRC stages using absolute gene set enrichment analysis (absGSEA). This methodology, integrated with experimental validation and survival analysis in a large independent cohort, provides a novel approach to stage-specific biomarker discovery.

Based on the enrichment analysis via absGSEA, some of the pathways identified include transcriptional co-regulatory mechanisms and protein kinase functional activity pathways enriched in adenocarcinoma compared to adenoma, exhibiting the advanced stage of CRC’s proliferative and aggressive nature [41,42]. The transcriptional co-regulatory pathway is critical in the invasive stage of CRC as it modulates the gene expression networks that support rapid cell division and survival under stress, while the protein kinase pathway, involved in signaling pathways including MAPK or PI3K/AKT, facilitates key oncogenic processes such as uncontrolled cellular growth, angiogenesis and metastasis [41,43]. In contrast, compared to adenoma, CIS showed pathways related to apoptotic processes and Wnt signaling pathways, suggesting mechanisms that may impact tumor development and progression [44,45]. Furthermore, the extracellular matrix (ECM) structural organization pathway was enriched in adenocarcinoma compared to CIS, highlighting ECM remodeling as a hallmark of advanced cancer. This ECM restructuring facilitates tumor cell migration and invasion, which is critical for metastasis [46,47].

The identified candidate biomarkers in this study have the potential for prognostic and diagnostic applications, as they represent unique activations, specific to each stage of CRC. Comparative analysis between adenoma with adenocarcinoma samples showed that *ARRB1*, *CTBP1* and *CTBP2* are overexpressed in adenoma. These genes were co-expressed and function as components of the transcriptional co-regulatory mechanism pathway. Additionally, they are also involved in other identified pathways, including transcription co-activator activity and transcription regulatory activity pathways. *ARRB1* (β-arrestin 1) plays a fundamental role in regulating immune responses and inflammation through the endothelin-1 receptor (ET-1R) and G protein-coupled receptors (GPCRs). These receptors affect the nuclear factor-kappa B (NF-κB) pathway, which is crucial in CRC tumorigenesis [48]. The NF-κB pathway plays a dual role in inflammation and innate immunity while also driving cancer activation and tumor progression [49]. Additionally, the involvement of *ARRB1* in the Wnt/beta-catenin signaling pathway, a hallmark driver of CRC and the most representative canonical signaling pathways [50], suggests its potential as an early biomarker for the development of adenomas [51]. *CTBP1* and *CTBP2* (C-terminal binding proteins) play critical roles in transcriptional repression and are frequently implicated in cancer progression. These proteins are known to repress tumor suppressor genes, thereby promoting cancer progression [52]. The overexpression of the *CTBP1/2* has been reported in various cancer types, such as prostate [53], leukemia [54,55], ovarian [56], breast [57] and colon cancer [58]. Evidently, *CTBP1* has been linked to several cancer hallmarks through transcriptional regulation such as increased cell survival, proliferation, migration/invasion and epithelial-to-mesenchymal transition (EMT). As previously observed in adenomas, these proteins may contribute to early tumorigenesis by regulating EMT, cellular proliferation and apoptosis [59], in which EMT and apoptosis are the most well-established cancer hallmarks. *CTBP1* has also been reported to be associated with adenomatous polyposis coli (*APC*), a tumor suppressor, suggesting a role in suppressing Wnt target gene expression. In CRC, mutations in *APC* that involve the *CTBP*–*APC* interaction lead to a dysfunctional Wnt signaling, driving cancer development [60,61].

In adenocarcinoma samples, key upregulated candidate biomarkers include *COL1A2*, *CEBPZ*, *MED10* and *PAWR*. *COL1A2* (collagen type I alpha 3 chain) is a key structural component of the ECM and may indicate its role in tumor progression and TME. Enrichment pathway analysis identified *COL1A2* as a key component involved in the extracellular matrix structural organization pathway, exhibiting significant upregulation in adenocarcinoma compared to CIS. In addition, the expression of *COL1A2* was significantly elevated relative to adenoma and CIS, making it an excellent diagnostic biomarker for the adenocarcinoma CRC stage. These findings align with previous studies, such as those highlighted by Yuan et al. [62], suggesting the overexpression of *COL1A2* is linked to poor prognosis, and there was evidence that *COL1A2* is positively correlated with immune infiltration. Moreover, it was reported that *COL1A2* is a potential biomarker for detecting adenocarcinoma [63,64]. The Enhancer Binding Protein Zeta (*CEBPZ*) is a key cellular protein involved in mediating responses to environmental stimuli. It shares functional pathways with *ARRB1* and *CTBP1*/*2*, including the transcriptional co-regulatory mechanism, transcription co-activator activity and transcription regulatory activity pathways. Furthermore, *CEBPZ* is co-expressed with *PAWR* and *MED10*. As a DNA-binding transcriptional activator, *CEBPZ* specifically regulates the heat-shock protein 70 (*HSP70*) promoter and plays a role in hematopoietic differentiation. Notably, TNMplot analysis in an independent CRC cohort revealed a significant upregulation of *CEBPZ* expression in tumor-stage CRC compared to normal and metastatic tissues. However, this observation requires further investigation, as it was not validated within the scope of the present study with RT-qPCR. *MED10* (Mediator Complex Subunit 10) works as a co-activator for DNA-binding transcription factors that facilitate RNA polymerase II activation. Research has established an association between aberrant expression of the *MED10* gene and advanced tumor stages, as well as diminished survival outcomes in bladder urothelial carcinoma patients. Dysregulation of *MED10* can potentially lead to abnormal cell proliferation, invasion and metastasis [65]. *PAWR* (Pro-Apoptotic WT1 Regulator) acts as a tumor suppressor gene with potent antitumor activity, inducing apoptosis of uncontrolled cell growth through both intracellular and extracellular mechanisms [66]. Specifically, it induces apoptosis through the Fas-mediated pathway and binds to glucose-regulated protein 78 (*GRP78*), thereby activating the extrinsic apoptotic pathway [66,67,68].

In CIS, *RPS3A* and *COL4A5* were notably enriched in CIS and may serve as transitional markers between benign and invasive CRC stages. Their overexpression supports a shift in tumor biology that precedes full invasion. *RPS3A* is involved in pathways related to apoptotic processes and cellular component of synapses, while *COL4A5* is involved in pathways related to cell junction organization and cellular component of synapses. Ribosomal Protein S3A (*RPS3A*) is part of the 40S ribosome unit, fundamental for protein synthesis. Beyond its established role, it has been associated with cell proliferation and tumorigenesis, implying a potential involvement in cancer [69,70]. While nominally significant, the expression of *RPS3A* was elevated in CIS compared to adenoma and adenocarcinoma. *COL4A5* (Collagen Type IV Alpha 5 Chain) is a structural protein in basement membranes of epithelial tissues. Overexpression of *COL4A5* may play a role in the ECM in cancer [71,72]. While no definitive biomarkers for CIS currently exist that are robust enough to clearly distinguish it from adenoma and adenocarcinoma, exploring immune cell signatures across CRC stages could provide a more comprehensive insight into their progression and differentiation. For example, unique immune subsets such as follicular helper T cells, gamma delta T cells, and activated mast cells could be explored. Interestingly, naïve B cells exhibit the highest activation in CIS, while resting mast cells are completely depleted. Follicular helper T cells, known for their critical role in supporting B-cell responses and antibody production, are significantly increased in CIS. Such increases in CIS might indicate a more active immune response aimed at controlling early-stage cancer [73]. Gamma delta T cells bridge innate and adaptive immunity and have potent antitumor properties [74] and will attempt to modulate the TME. Taken together, these suggest a more robust immune surveillance and response in the early stage of cancer. Further analysis using Kaplan–Meier survival plots suggests differing roles for *ARRB1*, *CTBP1* and *CTBP2* in advanced CRC. When these genes are highly expressed, it is suggestive of better survival outcomes. Their upregulation in adenomas suggests they may serve as early biomarkers or drivers of tumorigenesis. Similarly, the higher expression of *RPS3A*, which is associated with poorer survival, highlights its potential as a marker for the transition from CIS to the invasive stage, consistent with its upregulation in CIS observed in our study. Furthermore, the survival associations for *COL1A2*, *PAWR* and *MED10* suggest potential tumor-suppressive roles, supporting their roles in tumor progression as they are upregulated in adenocarcinoma samples in our analysis.

Our study’s primary aim was focused on early stages and on identifying stage-specific gene expression profiles during development of CRC. However, future studies could integrate CMS stratification into stage-specific analyses to enhance biomarker specificity and therapeutic applicability, ultimately moving toward more personalized management strategies for CRC. Indeed, the CMS framework classifies CRC into four subtypes (CMS1–CMS4), each with distinct transcriptomic profiles and clinical implications. Specifically, CMS1 is associated with immune activation and microsatellite instability [75,76]. CMS2 exhibits canonical WNT/MYC signaling pathway activation [77]. CMS3 is characterized by metabolic dysregulation, and CMS4 shows prominent mesenchymal features and stromal invasion [13,77,78,79,80]. Considering these molecular subtypes could further refine biomarker identification and provide deeper biological insights. Despite not explicitly stratifying our data according to CMS categories, several pathways and biomarkers identified here show potential correlations with established molecular features of specific CMSs. For example, the enrichment of extracellular matrix organization pathways and elevated expression of mesenchymal-related genes such as *COL1A2* in the adenocarcinoma samples might align with CMS4, known for its stromal remodeling and aggressive phenotype. Likewise, the observed activation of Wnt signaling pathways in CIS samples relative to adenoma may correlate with the canonical features of CMS2, emphasizing early activation of tumorigenic signaling cascades characteristic of this subtype. Nevertheless, direct correlations between our stage-specific findings and CMS categories require dedicated future analyses, ideally involving subtype-specific transcriptomic profiling and comparative analyses across different CRC stages. In addition, future studies should consider integrating mutational data such as *APC*, *KRAS*, *BRAF*, *PIK3CA* and *TP53* to determine whether specific genetic alterations correlate with the clustering patterns observed in the heatmap analysis of our study.

Compared to existing molecular tools for colorectal cancer staging—such as *APC*, *KRAS*, *BRAF*, *NRAS* and *PIK3CA* mutations—our study offers a novel transcriptomic perspective by identifying stage-specific gene expression signatures that distinguish adenoma, CIS and adenocarcinoma. While these commonly used biomarkers provide valuable prognostic or therapeutic guidance, they are not always effective in distinguishing between early stages of CRC progression. Our findings suggest that genes such as *ARRB1*, *RPS3A*, *COL4A5* and *COL1A2* may offer greater stage-specific resolution. Furthermore, these transcriptomic biomarkers have the potential to complement traditional TNM staging and histopathology, particularly in ambiguous or early lesions, thereby enhancing diagnostic precision and guiding more personalized treatment decisions.

While this study provides deep insights into the transcriptomic profiles and immune characterizations across different CRC stages, several limitations should be acknowledged. Firstly, the sample size may not entirely capture the genetic heterogeneity of the studied population, potentially reducing the generalizability of our findings. To the best of our knowledge, this study represents one of the initial efforts to comprehensively characterize transcriptomic changes across adenoma, CIS and adenocarcinoma within a population residing in the UAE. While the overall study population was ethnically diverse—including Emirati and non-Emirati patients—the current dataset does not allow direct ethnicity-based comparative analyses. Given that approximately 30% of the studied patients were Emirati nationals, future studies should specifically stratify and analyze Emirati and other Arab subpopulations separately to explore potential ethnic or population-specific molecular signatures. Additionally, a limitation in our study is the inclusion of five patients under the age of 50 years. Early-onset CRC often correlates with hereditary cancer syndromes, such as Lynch syndrome or familial adenomatous polyposis (FAP), characterized by distinct genetic backgrounds and transcriptomic profiles compared to sporadic CRC. Our current analysis did not differentiate between hereditary and sporadic CRC cases, potentially introducing variability. Therefore, future analyses should explicitly segregate younger patients and conduct targeted mutational profiling to identify hereditary syndromes, thereby refining transcriptomic biomarker analyses. Another limitation of this study is the absence of mutational profiling for the samples analyzed. The transcriptomic heterogeneity observed in the adenocarcinoma cohort—where some samples clustered closely with adenomas—underscores the molecular complexity of this stage. This overlap in gene expression profiles may be driven by mutations in key CRC driver genes such as *APC*, *KRAS*, *BRAF*, *PIK3CA*, or *TP53*, which are known to significantly influence tumor behavior and progression. Integrating such genomic data could provide deeper insight into the observed expression variability and help explain differences in clinical outcomes within tumor stages. Future studies should incorporate mutational analyses to clarify the relationship between genetic alterations and transcriptomic patterns, ultimately supporting the development of more personalized therapeutic strategies. Additionally, while immune cell signatures offer deeper insights into the TME, single-cell RNA sequencing may be required to better resolve the cellular heterogeneity at each stage.

In conclusion, this study represents a profile of transcriptomics analysis of adenoma, CIS and adenocarcinoma samples, identifying key differentially expressed genes and enriched pathways involved in CRC progression. Our findings highlight the importance of transcriptional co-regulatory mechanisms and ECM remodeling in tumor progression, as well as the potential predictive consequences of *ARRB1*, *COL1A2*, *MED10*, *RSP3A* and others. In addition, the observed immune cell characterization suggests distinct immunological responses at each stage. This insightful evidence may contribute to the development of targeted screening strategies and personalized therapeutic approaches.

## 4. Materials and Methods

**Figure 8:**
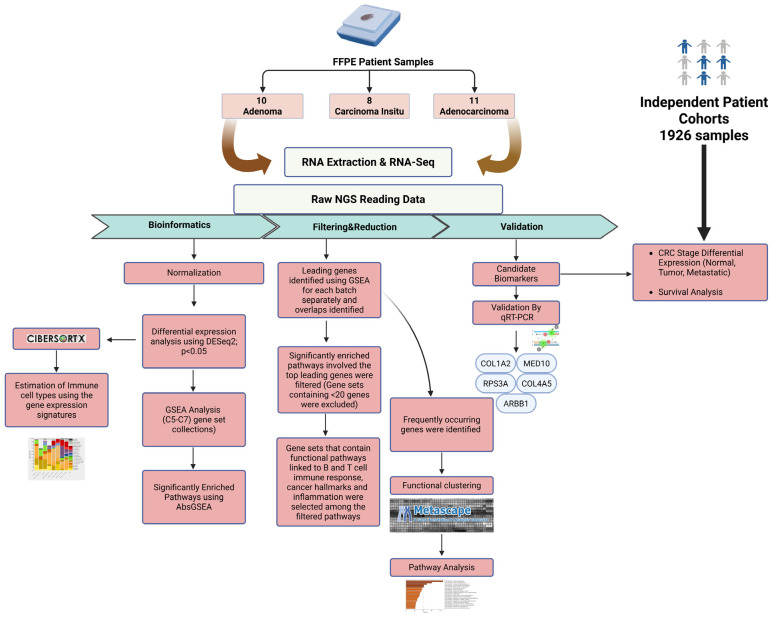
Flowchart outlines the steps of the bioinformatics and experimental approaches used to identify differentially expressed genes in adenoma, CIS and adenocarcinoma patient samples. The figure was created using BioRender.com (accessed on 5 December 2024).

The schematic diagram of the research methodology is given in Figure 8.

### 4.1. FFPE Tissue Specimens from Endoscopic Biopsies of CRC Patients and Independent Validation Cohort

This study involved a cohort of 29 patients, including 10 with tubular adenoma, 8 with carcinoma in situ and 11 with adenocarcinoma, all treated at the American Hospital Dubai (Table 2). Ethical approval was granted by the Dubai Scientific Research Ethics Committee (DSREC) of the Dubai Health Authority (DSREC-SR-02/2023_07). All procedures were carried out in accordance with the ethical standards outlined in the Declaration of Helsinki and the Belmont Report. Tumor staging and classification, including the assessment of tumor, lymph nodes and metastasis (TNM), were conducted under the supervision of two pathologists, K.S. and R.H. The included FFPE tissue biopsies were obtained from patients diagnosed with colorectal neoplasms and the cohort encompassed samples from both the colon and rectum. These were classified into three histopathological subtypes: adenoma, carcinoma in situ and adenocarcinoma. The inclusion criteria were as follows: adult patients with a confirmed histological diagnosis of colorectal adenoma, carcinoma in situ (CIS) or adenocarcinoma. All samples were obtained from patients without a history of inflammatory bowel disease (IBD), and each case represented a first-time diagnosis of colorectal neoplasia with confirmed pathological verification. As the primary objective of the study was to investigate stage-specific gene expression changes across the adenoma–CIS–adenocarcinoma progression, anatomical location (colon vs. rectum) was not used as a stratification criterion in the current analysis. To validate transcriptomic analysis findings, an independent CRC patient cohort comprising 1926 patient samples was analyzed through the TNMplot and KMplot platforms, with survival analysis conducted on 1336 patient samples to assess the prognostic relevance of the candidate biomarkers (further details in Section 4.8). These platforms integrate gene expression and clinical data from multiple publicly available sources including GEO (Gene Expression Omnibus), TCGA (The Cancer Genome Atlas), EGA (European Genome-phenome Archive) and GTex (Genotype-Tissue Expression Project), thereby representing diverse patient populations and geographic regions [81].

### 4.2. RNA Extraction

Three sequential sections, each 3 µm thick, from formalin-fixed paraffin-embedded (FFPE) tissue biopsies from the cases mentioned in Table 3 were subjected to RNA extraction using the Recover All kit according to the manufacturer’s instructions (Thermo Fisher Scientific, Waltham, MA, USA). Genomic DNA was removed from all RNA samples by treating the extracted RNA with the TURBO DNAase-free™ Kit (Invitrogen, Carlsbad, CA, USA).

Given the inherent limitations of FFPE samples, such as RNA degradation and technical variability that may affect RNA integrity, we employed the Qubit™ 3 Fluorometer (Life Technologies, Carlsbad, CA, USA) with the RNA HS Assay Kit to accurately assess RNA concentrations as it provides more reliable and sensitive results. Extracted RNAs were immediately used for sequencing. We also followed the recommended protocols for working with degraded FFPE RNA when preparing the libraries for RNA-seq. Subsequently, selected key DEGs were validated by RT-qPCR, confirming the consistency of expression trends across the three CRC stages. The consistent expression of the housekeeping gene GAPDH among samples was ensured.

### 4.3. Whole Transcriptome Sequencing

A total of 100 ng of extracted and Turbo DNase-treated RNAs was utilized for whole transcriptome sequencing from the 29 endoscopic biopsy samples. This was accomplished utilizing a targeted AmpliSeqTranscriptome Panel, meticulously designed to target over 21,000 human RNA transcripts through a high-throughput multiplexed approach on the Ion S5 XL Semiconductor Sequencer (Thermo Fisher Scientific, USA) equipped with the Ion 540 Chip (Life Technologies, Carlsbad, CA, USA). In summary, the total RNA underwent processing using the SuperScript VILO cDNA synthesis kit (Invitrogen; 11754050), followed by amplification of the resulting DNA using the lon AmeliSeq™ Transcriptome Human Gene Expression Kit (Thermo Fisher Scientific; A26325) for library preparation. Subsequently, the libraries underwent purification using Agencourt AM-Rute XP Beads (Beckman Coulter, Indianapolis, IN, USA) and quantification employing the lon Library TaqMan™ Quantitation Kit (Applied Biosystems, Waltham, MA, USA). The libraries were diluted to −100 pM and pooled by combining equal volumes of each barcoded library. The resulting template libraries were then sequenced on the Ion S5 XL Semiconductor sequencer using the Ion 540 Chip (Life Technologies Corporation, Carlsbad, CA, USA) prepared on the fully automated Ion Chef System (Thermo Fisher Scientific).

### 4.4. RNA-Seq Data Analysis

RNA-seq data analysis was performed using Ion Torrent Software Suite version 5.4 as previously described [82]. The raw reads generated were aligned to the hg19 (GRCh37) reference genome through the Torrent Mapping Alignment Program (TMAP), utilizing a two-stage mapping process to ensure high specificity and sensitivity. The raw read counts were normalized using the Fragments Per Kilobase Million (FPKM) method.

Principal component analysis (PCA) was conducted using R statistical software (version 4.2.0) with PCAtools package, and differential expression analysis (DEG) was performed with the DESeq2 package from R/Bioconductor. Raw read counts from the RNA sequencing data were used to identify differentially expressed genes (DEGs) across the endoscopic biopsy cohorts. Genes with fewer than 10 reads were excluded from further analysis. A significance threshold of *p* < 0.05 was applied to identify DEGs, prioritizing sensitivity in detecting probable biological signals, focusing mainly on genes associated with inflammation and immune response. An unadjusted threshold was applied to avoid strict filtering, which could eliminate biologically relevant genes, especially those with moderate expression changes. To improve the robustness of the findings, subsequent downstream gene set enrichment analysis (GSEA) was conducted to refine the DEG selection based on pathway-level significance, providing additional biological validation.

### 4.5. Gene Set Enrichment Analysis

The significant differentially expressed genes (DEGs) identified were examined to determine the activated and enriched cellular pathways related to various cancer stages using absolute gene set enrichment analysis (absGSEA) as described previously [27,82]. This analysis was conducted on the expression matrix using the absGSEA custom script written in R (v4.3.2), which computes sample-wise enrichment scores for each gene set. The complete R script and gene set inputs are available upon request to ensure full reproducibility. The full Molecular Signatures Database (MSigDB) includes approximately 90,000 annotated cellular pathways from the Broad Institute’s database (https://www.gsea-msigdb.org, accessed on 5 December 2024) along with custom pathways. A significance threshold of nominal *p*-value < 0.05 and False Discovery Rate (FDR) of *q*-value < 0.25 were taken into consideration to identify significantly activated pathways. Specifically, immunity and inflammation pathways were analyzed to identify differentially enriched genes across the endoscopic biopsy cohorts by performing leading-edge analysis, as described in [27]. The gene sets resulting from this process were refined by systematically cross-referencing each gene enriched in the statistically significant pathways. Lastly, genes that were commonly present across various enriched pathways were selected for further analysis.

### 4.6. Functional Enrichment Analysis by Metascape

Functional clustering and pathway analysis of the common DEGs or frequently occurring genes were conducted using Metascape (http://metascape.org, accessed on 17 September 2024) [42]. To validate the pathways identified through GSEA, the frequently occurring genes across all gene sets were further analyzed using Metascape for comprehensive pathway enrichment and functional categorization.

### 4.7. Validation of Selected Candidate Biomarkers Using Quantitative Reverse Transcriptase-PCR (RT-qPCR)

Complementary DNA (cDNA) was generated from 300 ng of total RNA using the High-Capacity cDNA Reverse Transcription Kit (Applied Biosystems, Waltham, MA, USA), which employs a combination of random hexamers and oligo(dT), following the manufacturer’s protocol. Gene expression was analyzed by quantitative PCR (qPCR) with Maxima SYBR Green/ROX qPCR MasterMix (2×) (Thermo Fisher Scientific) on a QuantStudio3 Real-Time PCR thermal cycler (Applied Biosystems). The qPCR was conducted using the primer sets listed in Table 3. *GAPDH* and *18S* were used as housekeeping genes. The relative expression levels of the target gene were calculated using the 2^−ΔΔCt^ method relative to the *GAPDH* gene.

### 4.8. Validation of Putative Biomarkers in Independent CRC Patient Cohorts via TNMPlot, KMplot and cBioPortal

The putative biomarkers derived from the RNA-seq were validated using independent cohorts through three different analyses. Identified genes through absGSEA analysis were analyzed for different CRC types, including adenoma (*ARBB1*, *CTBP1*, *CTBP2*), adenocarcinoma (*COL1A2*, *CEBPZ*, *MED10*, *PAWR*) and CIS (*COL4A5*, *RPS3A*). (a) Gene expression analysis: comparison of expression levels of candidate genes was assessed across normal, tumor and metastatic tissues by using the TNMPlot database in an independent cohort comprising 1926 samples (https://tnmplot.com/; accessed on 5 December 2024). (b) Survival analysis was completed by using the Kaplan–Meier Plotter (https://kmplot.com/; accessed on 5 December 2024) on an independent CRC patient cohort consisting of 1336 patients to generate the overall survival (OS) curves for some of the key genes identified after absGSEA analysis for adenoma, adenocarcinoma and carcinoma in situ cohorts.

### 4.9. Exploration of Immune Cell Characteristics

Cell-Type Identification by Estimating Relative Subsets of RNA Transcripts (CIBERSORTx) was employed to identify immune cell signature profiling for each CRC stage (https://cibersort.stanford.edu, accessed on 5 December 2024). The algorithm uses gene expression signatures composed of approximately 500 genes. We utilized the LM22 gene signature file, which contains 547 genes and distinguishes 22 human hematopoietic cell phenotypes, including 7 T cell types, naïve and memory B cells, plasma cells, NK cells, and myeloid subsets. Normalized gene expression data from adenoma, adenocarcinoma and carcinoma in situ cohorts, were uploaded to the CIBERSORTx web portal for analysis.

## 5. Conclusions

The identified candidate biomarkers represent distinct stages in CRC development, from adenoma to carcinoma in situ to ultimately adenocarcinoma. Notably, CIS was characterized by significant enrichment of apoptotic processes and Wnt signaling relative to adenoma, while adenocarcinoma revealed significant pathway enrichment in transcriptional co-regulatory mechanisms compared to adenoma. Additionally, extracellular matrix organization and remodeling was primarily enriched in adenocarcinoma compared to CIS. Their roles emphasize critical pathways that are fundamental in CRC progression. Among the identified molecular targets, *ARRB1* was validated using RT-qPCR for adenoma, *COL4A5* and *RPS3A* for CIS, and *COL1A2* and *MED10* for adenocarcinoma. Further functional studies are necessary to validate their potential as diagnostic and therapeutic targets as well as to explore their mechanistic roles. While further validation in larger, independent cohorts is warranted, the current findings provide a foundation for future translational research.

## Figures and Tables

**Table 1:** Gene sets used from the MSigDB database and their characteristics.

Gene Sets Used	Type
C1	Positional Gene Sets
C2	Curated Gene Sets
C3	Regulatory-Related Gene Sets
C4	Computational Gene Sets
C5	Gene Ontology Gene Sets (Biological Processes, Molecular Functions, and Cellular Components)
C6	Oncogenic Related Gene Sets
C7	Immunology-Related Gene Sets
C8	Cell type Signature Gene Sets

**Table 2:** Patient characteristics for the 29 biopsies collected from adenoma, CIS and adenocarcinoma patients in the UAE. NA: Not Applicable.

No	Gender	Age	Nationality	Subtype
1	Male	61	Italian	Tubular Adenoma
2	Male	61	Qatari	Tubular Adenoma
3	Male	54	Emirati	Tubular Adenoma
4	Male	61	Emirati	Tubular Adenoma
5	Female	39	Italian	Tubular Adenoma
6	Male	75	Indian	Tubular Adenoma
7	Female	64	British	Tubular Adenoma
8	Male	48	Portuguese	Tubular Adenoma
9	Female	51	Emirati	Tubular Adenoma
10	Male	50	South African	Tubular Adenoma
11	Female	59	French	Adenocarcinoma
12	Female	43	Filipino	Adenocarcinoma
13	Male	56	Swiss	Adenocarcinoma
14	Female	64	Emirati	Adenocarcinoma
15	Female	38	Iraqi	Adenocarcinoma
16	Female	44	Sudanese	Adenocarcinoma
17	Female	32	Emirati	Adenocarcinoma
18	Female	65	Egyptian	Adenocarcinoma
19	Male	77	Indian	Adenocarcinoma
20	Male	84	Syrian	Adenocarcinoma
21	Female	65	Egyptian	Adenocarcinoma
22	Male	70	Romanian	Carcinoma in situ
23	Female	80	Emirati	Carcinoma in situ
24	NA	NA	NA	Carcinoma in situ
25	Male	NA	Filipino	Carcinoma in situ
26	Female	NA	Emirati	Carcinoma in situ
27	Male	NA	Emirati	Carcinoma in situ
28	Female	NA	Egyptian	Carcinoma in situ
29	NA	NA	NA	Carcinoma in situ

**Table 3:** Sequence of primer pairs used in the qPCR.

Gene ID	Forward Primer Sequence	Reverse Primer Sequence
*COL1A2*	TCAAAGGCATTAGGGGACACA	CATTTTCACCAGGGGCACCA
*CEBPZ*	GAAGAGTTTGGCCATCTATTGG	CATCTAAGCTGTTTGAGACTTG
*MED10*	AGATCGACACCATGAAGAAAT	TGAGCTGGTTAAGAAGGCGG
*PAWR*	GCGGAAACGAGAAGATGCAA	TTCTAGGTGGCTCCTGTAG
*ARRB1*	GCGGAGAGTCTATGTGACG	ACAGGTCCTTGCGAAAGGT
*CTBP1*	GATGTCAGGCGTCCGACC	ACAGTGGCCACGTCCTTCA
*CTBP2*	AATCCCCCACAGCGGTCCA	GTGACGCCACTATGAACCTG
*RPS3A*	GGACCCAAGGAACCAAAATT	AGTTTTTACCCTGAACATCTTC
*COL4A5*	TCCCAAAGGATTACCAGGCA	GGAACCCAACTCTCCTTTGT
*18S*	TGACTCAACACGGGAAACC	TCGCTCCACCAACTAAGAAC
*GAPDH*	TGTCAGTGG TGGACCTGACCT	TCGCTGTTGAAGTCA GACGAG

## Data Availability

The RNA sequencing data generated in this work have been deposited in the Gene Expression Omnibus (https://www.ncbi.nlm.nih.gov/geo (accessed on 17 September 2024)) under GEO Series access number GSE266390 and can be accessed from https://www.ncbi.nlm.nih.gov/geo/query/acc.cgi?acc=GSE266390 (accessed on 17 September 2024). All other supporting data of this study are either included in the manuscript or available on request from the corresponding author.

## Supplementary Materials

The following supporting information can be downloaded at: https://www.mdpi.com/article/10.3390/ijms26094194/s1.
