# Supplementary figures and images for: Transcriptome-Wide Analysis and Experimental Validation from FFPE Tissue Identifies Stage-Specific Gene Expression Profiles Differentiating Adenoma, Carcinoma In-Situ and Adenocarcinoma in Colorectal Cancer Progression
Source: Int J Mol Sci. 2025 Apr 28;26(9):4194. doi: 10.3390/ijms26094194 (PMC12071244; doi:10.3390/ijms26094194)

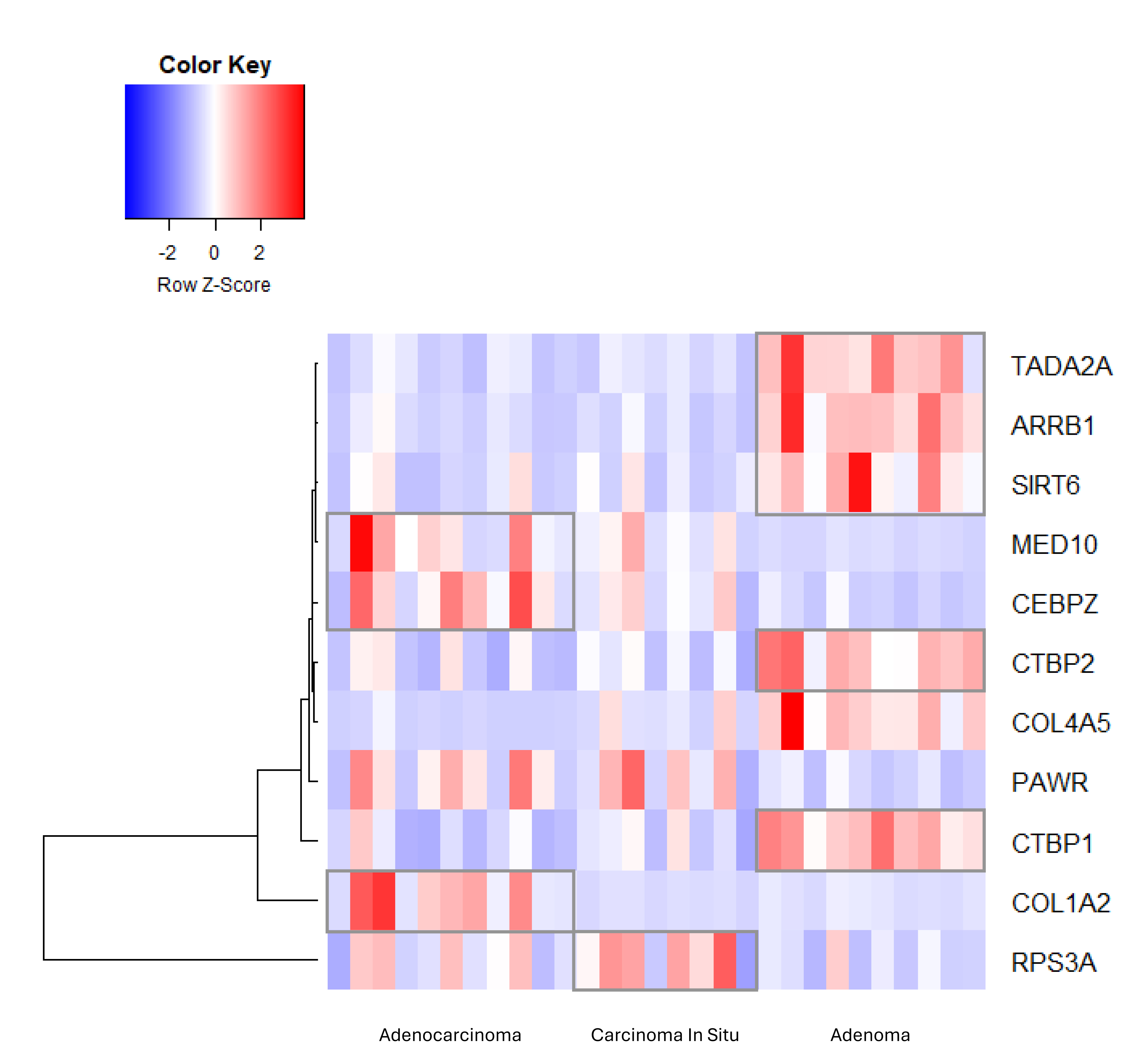

Supplement: Supplementary file 1 [file ijms-26-04194-s001.zip › Figure S1.jpg]
